# Impacts of Land Cover Data Selection and Trait Parameterisation on Dynamic Modelling of Species’ Range Expansion

**DOI:** 10.1371/journal.pone.0108436

**Published:** 2014-09-29

**Authors:** Risto K. Heikkinen, Greta Bocedi, Mikko Kuussaari, Janne Heliölä, Niko Leikola, Juha Pöyry, Justin M. J. Travis

**Affiliations:** 1 Finnish Environment Institute, Natural Environment Centre, Helsinki, Finland; 2 Institute of Biological Sciences, University of Aberdeen, Aberdeen, United Kingdom; Institute of Botany, Czech Academy of Sciences, Czech Republic

## Abstract

Dynamic models for range expansion provide a promising tool for assessing species’ capacity to respond to climate change by shifting their ranges to new areas. However, these models include a number of uncertainties which may affect how successfully they can be applied to climate change oriented conservation planning. We used RangeShifter, a novel dynamic and individual-based modelling platform, to study two potential sources of such uncertainties: the selection of land cover data and the parameterization of key life-history traits. As an example, we modelled the range expansion dynamics of two butterfly species, one habitat specialist (*Maniola jurtina*) and one generalist (*Issoria lathonia*). Our results show that projections of total population size, number of occupied grid cells and the mean maximal latitudinal range shift were all clearly dependent on the choice made between using CORINE land cover data vs. using more detailed grassland data from three alternative national databases. Range expansion was also sensitive to the parameterization of the four considered life-history traits (magnitude and probability of long-distance dispersal events, population growth rate and carrying capacity), with carrying capacity and magnitude of long-distance dispersal showing the strongest effect. Our results highlight the sensitivity of dynamic species population models to the selection of existing land cover data and to uncertainty in the model parameters and indicate that these need to be carefully evaluated before the models are applied to conservation planning.

## Introduction

One of the challenges in conservation and management planning is developing robust assessments of the impacts of climate change on species’ ranges. To date, such assessments have relied on static ‘bioclimatic envelope’ (‘BEMs’), or ‘environmental niche’ models (‘ENMs’) [1], [2], which relate the species’ distributions to current climate and then project future ranges by fitting the derived models to different climate scenarios. However, the capacity of BEMs to provide useful guidelines for climate change oriented conservation planning is limited. First, their outputs are rather coarse-scaled and provide little understanding of potential differences in species’ responses in different parts of the study region [3], [4]. Second, BEMs generally do not account for the fact that a species’ range expansion depends on the characteristics of the landscape over which individuals disperse [5]–[7]. Importantly, connectivity of the habitat network has a critical role in species’ range dynamics [8], [9].

Dynamic models for species’ range shifts are a promising tool for conservation biology providing improved possibilities for assessing species’ abilities to track the changing climate and persist in a habitat network [6], [10], [11]. There are a few example of such models, with applications to habitat networks developed at local [12],[13], regional [3], [14], [15] and national scale [16]. However, although these models hold much promise, they have potential caveats which need to be explored to avoid their uncritical implementation [12], [17], [18].

In this study we address two potentially important sources of uncertainty: the selection of habitat maps and the parameterisation of species’ life-history traits. Modelling studies conducted over large areas face the challenge of obtaining sufficiently robust data on the distribution of suitable habitats for the species [19]. This is because more accurate spatial data on species’ habitats are often available only for some intensively surveyed localised areas. Land cover databases gathered over large areas, such as national CORINE databases in Europe, rely often on remote sensing and other data sources and are thus likely to show substantially more within-land-cover-type variation in habitat quality than the habitat maps based on intensive field surveys. Recent studies suggest that this lack of fine resolution in habitat classification is a feature of European-wide databases including CORINE 2000 and 2006 and that this may cause biases in modelling [20]. A key problem is that coarse resolution classification can result in larger areas being classified as suitable habitat for a species than there is in reality, especially in the case of habitat specialists. This topic has been surprisingly poorly investigated in the context of models for projecting range expansion, although a few exceptions [12], [16] suggest that varying the amount of habitat in the landscape can have a significant impact on the outputs of dynamic models.

A second main challenge and source of uncertainty is to develop accurate estimates for species’ dispersal abilities and demographic parameters [21]–[23]. The confidence in the species’ parameters employed in simulations for range expansion is often very limited. Thus, to be useful for conservation, dynamic simulation models should provide estimates of the extent to which model outputs are sensitive to these uncertainties. Sensitivity analyses provide means for addressing this problem and for giving more robust confidence intervals to the projections. A number of studies employing simulated landscapes have shown that projections of species’ expansion rates may be rather sensitive to the parameter values for certain key life-history traits [6], [24], [25]. However, corresponding studies carried out for real species on real landscapes have addressed model sensitivity to various degrees. Some of them have scrutinised the impact of varying several species’ parameter values [12], [15], [16], while others have assessed the model sensitivity to only one or very few life-history parameters [e.g. 13], referred to a priori tests [e.g. 14], or otherwise provided limited information on the sensitivity of model projections to species’ parameter selection [e.g. 26].

Here we investigate the impacts of land cover data selection and parameterisation of species’ traits on the projected species’ range expansion dynamics by using two butterfly species (*Maniola jurtina* and *Issoria lathonia*) inhabiting different types of grasslands in Finland. Butterflies are useful model species for studying range expansion and ecological sufficiency of habitat networks because they have the potential to respond rapidly to climate change [27]–[29]. Our focal study environment, unimproved grasslands, represents important habitat for nature conservation throughout Europe [30], [31]. These habitats are threatened due to agricultural intensification and abandonment of marginal areas [32]–[34], which is likely to hamper the range expansion of grassland specialist species [28], [35].

Our main objective in this study is to compare the degree of uncertainty associated with the land cover data selection with that stemming from the species’ life history parameterisation. The range dynamics and population persistence capacity of our two example butterfly species is explored using RangeShifter, a novel dynamic modelling platform for species’ range dynamics [36]. Both species are reliant in Finland on the network of grassland biotopes. We constructed representations of this grassland network using two different extensive sources of land cover and land use data sets. The first data set is the European-wide CORINE land cover database, while the second data set is a combination of three sources, the National Survey of Valuable Traditional Rural Biotopes, grassland sites managed based on the Agri-environment scheme (AES) [37], [38], and data on distribution of all types of grasslands in Finland, gathered in the SLICES land cover database [39]. For species traits, we focused on the separate impacts of four key dispersal and demographic parameters which are likely to affect the model outcomes: population growth rate, carrying capacity, mean dispersal distance and probability of long-distance dispersal events [6], [12], [16], [40].

## Materials and Methods

### Study species

Our two model species were Meadow Brown *Maniola jurtina* (Lepidoptera, Nymphalidae) (Linnaeus, 1758) and Queen of Spain Fritillary *Issoria lathonia* (Lepidoptera, Nymphalidae) (Linnaeus, 1758). *Maniola jurtina* is a grass-feeding species which behaves as a grassland habitat specialist in Finland, where it occurs at its northern range boundary [cf. 41]. In the butterfly transect monitoring surveys in Finland, the species has been found to favour managed (dry) unimproved grasslands over other types of grasslands [42]. We acknowledge that the species is a common grassland generalist in other parts of Europe, especially areas south of Finland [43]–[45]. In contrast, *Issoria lathonia* is a violet-feeding generalist fritillary and behaves in Finland similarly as in other parts of Europe [46]. It is capable of inhabiting many different grassland types, including lower quality grasslands such as set-asides and grassy strips along field margins [43], [47], [48]. We focus on these two butterfly species because they provide useful examples of ecologically contrasting species inhabiting the grassland habitat network. Moreover, the current range of both species is limited to southern Finland, from where they can be expected to move northwards following the warming climate, making them realistic model species for simulating range expansion dynamics.

**Figure 1:**
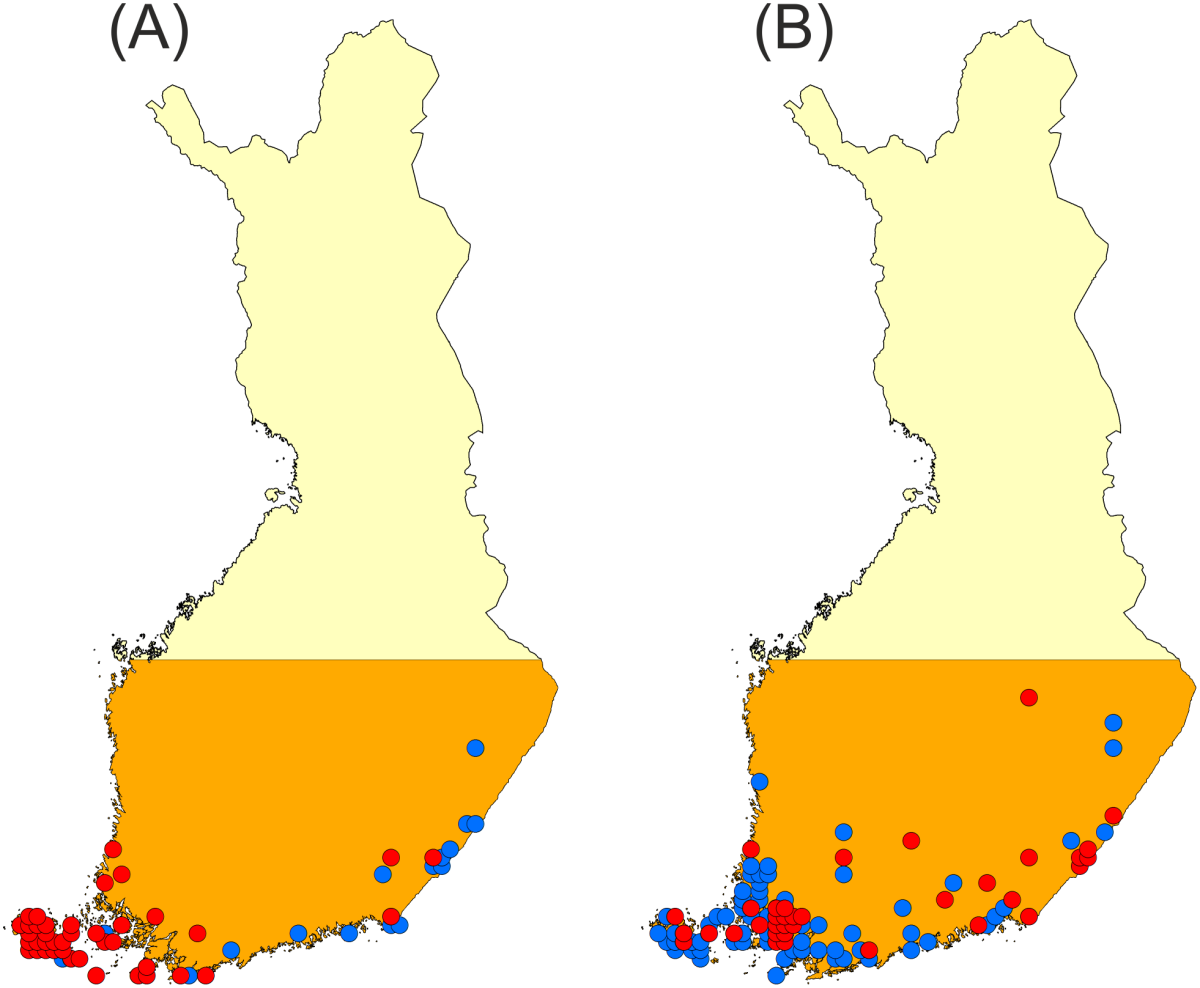
10×10 km grid cells with known occurrences for (A) *Maniola jurtina* and (B) *Issoria lathonia*. The occurrence records in the 10×10 km grid cells in Finland were divided into the two time periods, 1991–2000 (red dots) and 2001–2011 (blue dots). Area where the range expansion simulations were performed is shown with orange.

The known occurrence records for the two study species were extracted from the National Butterfly Recording Scheme in Finland (NAFI). The NAFI is based on observations made by professional and volunteer amateur lepidopterists using a uniform 10×10 km grid system for the whole country [49], [50]. We divided these records into two time periods, 1991–2000 and 2001–2011, and used the data from the first period, 1991–2000, to select the areas for initialising the simulations (i.e. the 10×10 km with records of species occurrences; see Figure 1). It should be noted that the butterfly occurrence records for the whole study area (Figure 1) were available only at this resolution although solitary records have been made using finer resolution mapping. Therefore, in our simulations, we were constrained to initialise the butterfly populations at a resolution of 10×10 km. Moreover, as all the simulations were run at a of 200×200 m (see below), all the 200×200 m cells included in a 10×10 km with known occurrence records were seeded.

### Land cover data

The first main source of land cover data employed was the CORINE 2000 Land Cover database. We opted for these data due to their complete spatial coverage of Finland, because of the shared methodology at the pan-European level (EU countries) and because they are widely used in studies on impacts of land use on species distributions [e.g. 51,52]. The classification of land cover into CORINE classes in Finland is based on automated interpretation of Landsat ETM satellite images and subsequent data integration with existing digital maps on land use and soil information [53]. The resolution of the CORINE data is 25×25 m in Finland and the classification of land cover includes four hierarchical levels. However, as we ran our simulations at a resolution of 200×200 m, we scaled-up the CORINE data for the relevant land cover types by simply summing-up their cover based on the sixty-four 25×25 m resolution cells embedded in each of the 200×200 m grid cells. This was done throughout the study area, which comprised southern Finland up to approximately the latitude of 63°N (Figure 1).

For the grassland specialist species, *Maniola jurtina*, we calculated the cover at 200×200 m resolution of the CORINE categories ‘Pastures’ (2.3.1 in CORINE classification) and ‘Natural grassland’ (3.2.1 in CORINE). For *Issoria lathonia*, the grassland generalist, the CORINE categories 2.4.3 (‘Land principally occupied by agriculture, with significant areas of natural vegetation’) and 2.1.1.2 (‘Abandoned arable land’) were additionally included, together with field margins measured based on the CORINE class 2.1.1 (arable land), when assessing the total amount of suitable habitat. Field margins’ cover was estimated by multiplying their length in a 200×200 m cell by an effective width of 1 meter, based on empirical observations from monitoring schemes of grassland butterflies. For both the study species, a given 200×200 m grid cell was considered to be potentially suitable “habitat” for the species if it contained some amount of the above listed CORINE classes (thus there was no threshold for the amount of particular type of grassland required for a 200×200 m cell to be considered habitat). The percentage habitat cover determined the cell total carrying capacity for each species. For example, the carrying capacity *K* for *Issoria lathonia* was estimated to be approximately 60 individuals/ha (see *Species parameterisation and dynamic range expansion modelling*); thus for a 200×200 m cell with 100% cover of suitable habitat, the maximal potential total carrying capacity was 240 individuals (for more details see [36]).

Simulations conducted with the CORINE-based grassland network were compared with those ran using more detailed data for grasslands, obtained by combining three different national grassland databases. For *Maniola jurtina*, we calculated the summed cover of all open grasslands in each of the 200×200 m grid cells mapped in (1) the National Survey of Valuable Traditional Rural Biotopes [54], together with the cover of open grassland sites included in (2) the national Agri-environment scheme (AES) in Finland [see 39]. Because both National Survey and AES-based managed grasslands initially included also wooded sites, we used (3) the SLICES land cover database to dissect the open grasslands from the wooded ones [39]. The SLICES database, which is compiled by the National Land Survey of Finland, shows the distribution of all types of common treeless grasslands in Finland. For *Issoria lathonia*, we added the non-overlapping SLICES grasslands to the open AES-managed grasslands and National Survey grasslands, in order to construct an estimate of the total habitat available for a grassland generalist species. All the habitat analyses and calculations were done by using ArcView Spatial Analyst (Version 3.2, ESRI Inc., Redland, CA, USA).

Additionally, for illustrative purposes we calculated the difference in the amount of suitable habitat estimated with the two approaches (i.e. the CORINE database vs. the AES-National Survey-SLICES databases). For this, we calculated for each of the 10×10 km grid cells included in our study area, the amount of habitat classified as suitable using each of the two methods, and report the spatial distribution of differences between the two methods.

### Species parameterisation and dynamic range expansion modelling

Range expansion modelling was conducted using RangeShifter v1.0, a platform for individual-based dynamic modelling of single species’ ecological and evolutionary dynamics [36]. At the heart of RangeShifter is the explicit modelling of population dynamic and dispersal, the latter divided into its three fundamental phases of emigration, transfer and settlement.

From the options available within RangeShifter we chose to use a female-only and non-overlapping generations population model [55], which requires the population intrinsic growth rate (*R_max_*) and carrying capacity (*K*). We assumed one reproductive season per year [cf. 56]. After reproduction all adults die and each offspring have a density-dependent probability of dispersing given by the following equation:
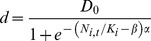
where *D_0_* is the maximum emigration probability, *β* is the inflection point and *α* is the slope of the curve at the inflection point. *N_i,t_* represents the number of individuals in cell *i* at time *t*, while *K_i_* is the carrying capacity of the cell. We fixed the above parameters to *D_0_* = 0.4, *β* = 1.0 and *α* = 5.0 (for the derived emigration probability curve see Figure S1). For those individuals who disperse, the distance is sampled from a double negative exponential distribution [57], [58]. This is composed from two negative exponential distributions with different means and different probabilities of occurrence: the first, more common and with shorter mean (‘dispersal I’); the second, less common and with longer mean in order to simulate relatively rare and long distance dispersal events (‘dispersal II’). The dispersing individual is displaced at the sampled distance in a random direction. If the arrival cell is unsuitable, the individual is either displaced in one of the eight nearest neighbouring cells, if any of those is suitable, or assumed to die. We assume no additional dispersal mortality.

We conducted an extensive literature search to specify values for the model parameters for our study species. To supplement information from the literature, we used data extracted from long-term butterfly monitoring surveys carried out in Finland, in particular the transect count data from the Finnish Butterfly Monitoring Scheme [59], as well as expert-knowledge-based assessments on the study species’ biology. Where required, dispersal and demographic parameter values were further adjusted based on studies on ecologically similar species and, more generally, on how life-history traits have been observed or estimated to vary among grassland butterfly species [cf. 14,60]. A more in-depth description of the study species’ parameterisation process is included in Text S1. Following we provide the key information.

For the four focal life-history parameters in our model, i.e. carrying capacity *K*, maximum population growth rate *R_max_*, mean dispersal distances and probability of long-distance dispersal events, an intermediate ‘default’ value and lower and higher alternative values were determined for both of the model species. We estimated carrying capacity from the data from the Finnish Butterfly Monitoring Scheme [59] and selected literature [43]. *K* = 250 individuals/ha was employed as carrying capacity value, while *K* = 200 and *K* = 300 as the lower and higher alternative for *Maniola jurtina*, and *K* = 60 individuals/ha as default value and *K* = 30 and *K* = 90 as the two alternatives for *Issoria lathonia*. The amount of grassland habitat deemed suitable for the study species was employed to assess the maximal potential size of the population in each of the 200×200 m cells which were either initially seeded or modelled to be colonised during the simulation, and this assessment was conducted based on the three different values of *K* for both the species (lower alternative for *K* resulted in lower estimates of 200×200 cell population size, and higher alternative to higher estimates, respectively).

The population growth rates were determined based on measurements on ecologically similar (and dissimilar) butterfly species from the literature, and expert judgements based on field observations. These suggested that both *Maniola jurtina* and *Issoria lathonia* are likely to show intermediate population growth rates. Thus, we used *R_max_* = 2.0 as the default value and *R_max_* = 1.5 and *R_max_* = 2.5 as the two alternatives for both species [15].

We used only one value for the mean short-distance dispersal distance: 150 m for *Maniola jurtina* [21], [61]–[64], and 300 m for the more mobile *Issoria lathonia* [47], [62], [65], [66]. For the mean long-distance dispersal distance, we used 3 km as the intermediate default value and 1.5 and 5 km as alternatives for *Maniola jurtina,* and 3, 5 and 10 km for *Issoria lathonia*. Based on the observations of Öckinger and Smith (2007) [62] on *Maniola jurtina* movements, we set the probability of individuals dispersing with the first, short distance dispersal kernel to either 0.80, 0.90 (default) or 0.95.

We assumed no environmental stochasticity because our focus was examining the potential impacts of the four key species life-history traits on range expansion simulation results. However, it should be noted that RangeShifter inherently incorporates two other key sources of stochasticity, demographic stochasticity and stochasticity in dispersal [67], [68].

Species distribution data for the years 1991–2000 were used as a starting point for the simulations. All the 200×200 m grid cells with some suitable grassland habitat and located in the occupied 10×10 km species’ distribution cells were initialised with a number of individuals equal to the cell total carrying capacity, determined by the habitat percentage cover in the cell. This initialisation approach very likely produced an exaggerated abundance for the species as a starting point. However, pilot runs showed that there was only a 2–5 year burn-in phase in the simulations during which the initialised cells with too little habitat or too isolated in space lost their individuals, after which the total simulated population size either remained constant or started to increase. All the simulations were run over a 50-year time window.

Varying the parameters as described above resulted in 9 different simulations for both of the study species which were conducted on the two alternative landscape maps. For each simulation, 100 replicate runs were conducted. Here we focus on the simulation results dealing with the species’ projected ranges: (1) total numbers of individuals, (2) total numbers of occupied cells and (3) species’ range extents and projected shifts in range, measured as the maximum latitude at the year 0 vs. the maximum latitude year 50 (east–west range shifts were not examined).

## Results

### Mapping habitat suitability using the alternative land cover data

**Figure 2:**
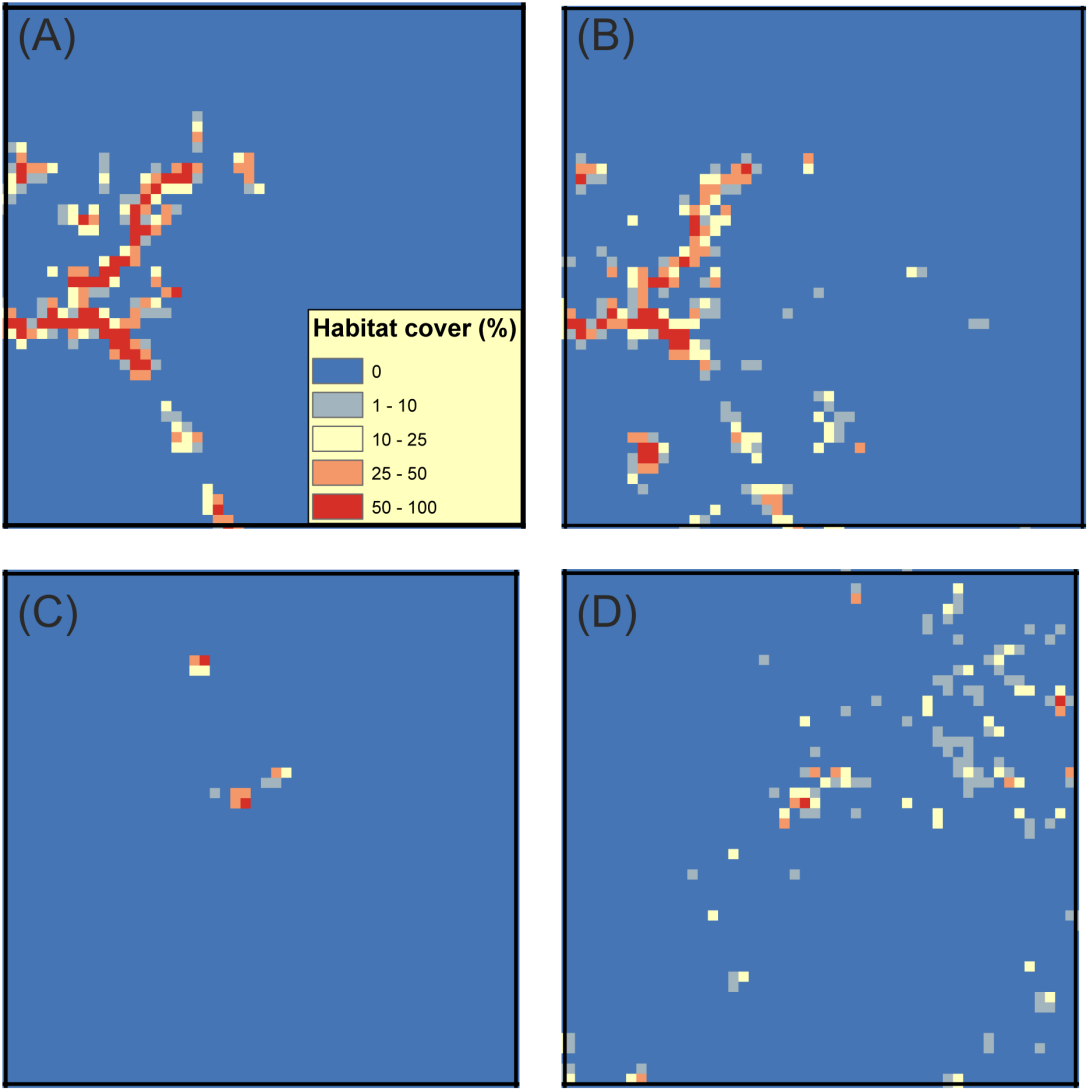
Variation in the estimated amount of suitable grasslands for *Maniola jurtina*, a grassland specialist butterfly. The amount of suitable habitat is shown for two exemplary 10×10 km grid cells and it was calculated based of the two different sources of grassland data. A–B: the first example 10×10 km cell; C–D: the second example 10×10 km cell. A and C: summed cover of open grasslands included in the National Survey and grasslands managed via Agri-environment Scheme (AES) in the 200×200 m cells; B and D: summed cover of CORINE classes ‘Pastures’ and ‘Natural grassland’ in the 200×200 m cells.

**Figure 3:**
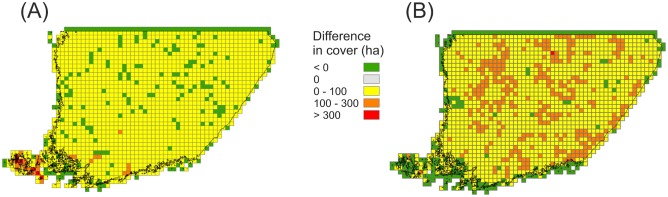
Difference in the amount of estimated suitable grasslands between the two land cover datasets. The distribution of differences was calculated by subtracting the amount of open grasslands in the National Survey-AES(-SLICES) databases from the amount of CORINE land cover types deemed as suitable for the two study species. The differences are shown in hectares across the 10×10 km grid cells of the simulation area. (A) *Maniola jurtina*: National Survey-AES grasslands were subtracted from the summed cover of the CORINE types ‘Pastures’ and ‘Natural grassland’; (B) *Issoria lathonia*:, National Survey-AES-SLICES grasslands were subtracted from the CORINE types ‘Pastures’, ‘Natural grassland,‘Land principally occupied by agriculture, with significant areas of natural vegetation’, ‘Abandoned arable land’ and field margins measured based on the CORINE class ‘arable land’.

Comparison of the total amount of suitable grassland habitat in the study area, i.e. the CORINE database vs. the AES-National Survey-SLICES databases, revealed substantial differences in both the studied butterfly species. These differences were greater for *Maniola jurtina,* the grassland specialist. In total, 7,705 ha are classified as suitable for *Maniola jurtina* when using AES-National Survey while it increases to 33,951 ha when using the CORINE database. For *Issoria lathonia* there is again greater total amount of suitable habitat when using CORINE database compared to AES-National Survey-SLICES databases (160,075 ha versus 60,035 ha). In addition, there is substantial spatial variation in the extent of the difference obtained using the two alternative datasets. An illustration for *Maniola jurtina* for two 10×10 km example grid cells shows one area where the CORINE-based habitat availability pattern is broadly similar to those based on data from AES-managed grasslands and grasslands included in the National Survey (Figure 2A vs. B), and another area where the CORINE data suggests that much more suitable habitat occurs in the landscape than AES - National Survey data (Figure 2C vs. D). Figure 3A illustrates the overall distribution of differences across all 10×10 km cells of the study region in Southern Finland. Greatest differences occur in SW archipelago where much more of the landscape is designated as suitable when using CORINE. A similar general pattern in differences (though more subtle) is found for *Issoria lathonia* in the two 10×10 km example grid cells (Figure S2), but the areas where the differences in the 10×10 km grid cells are greatest occur now in different regions (Figure 3B).

### Maniola jurtina – the grassland specialist

**Figure 4:**
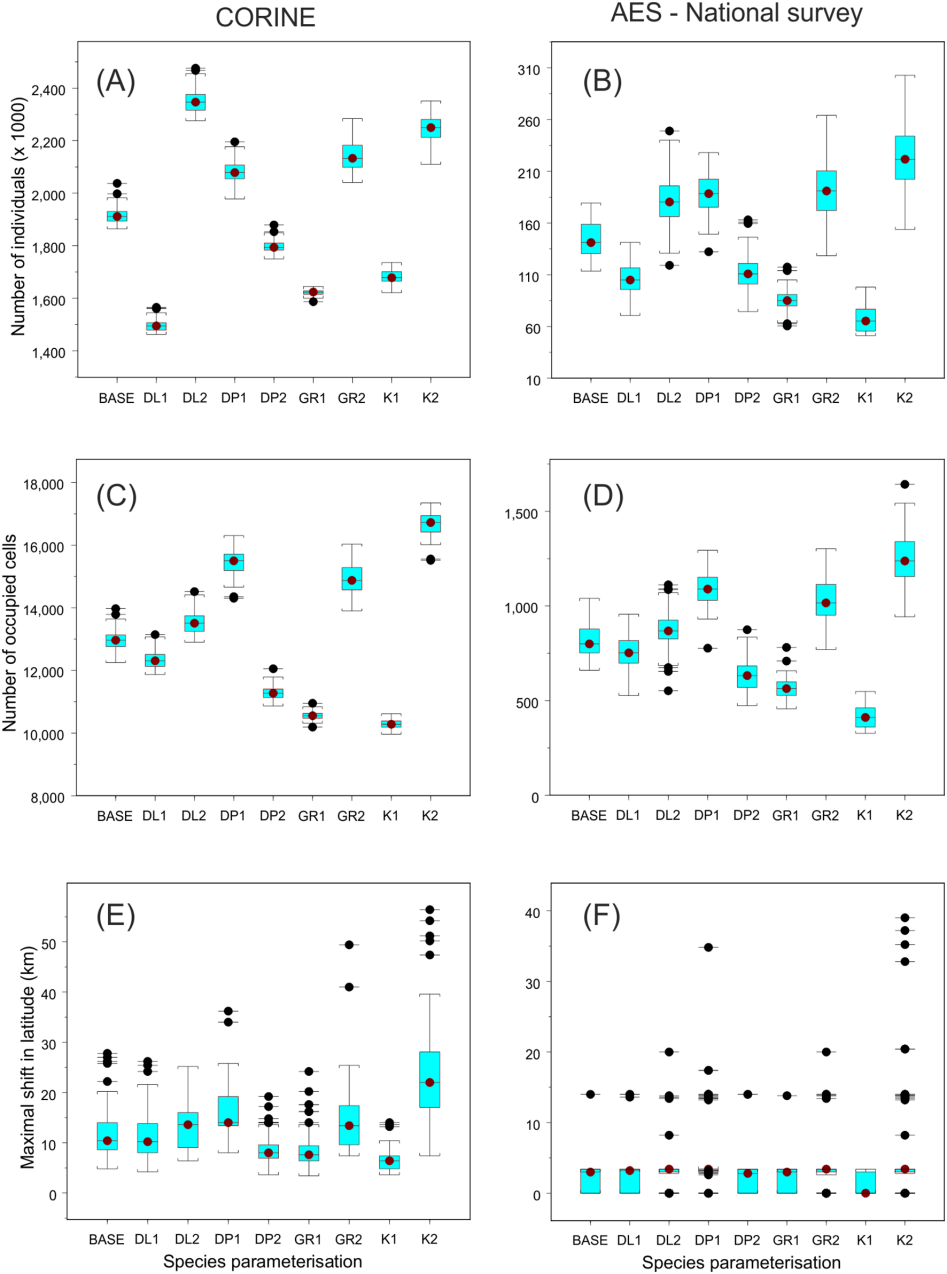
The total population abundance and range dynamic statistics for *Maniola jurtina.* The statistics include projected total number of *Maniola jurtina* individuals (A–B), number of 200×200 m cells occupied (C–D) and maximal range shift of the butterfly (E–F) at the end of a 50 year dynamic simulation period. Simulations were conducted using RangeShifter, a dynamic and individual-based modelling platform, and either summed cover of CORINE classes ‘Pastures’ and ‘Natural grassland’ (A, C, E) vs. open grasslands included in the National Survey of Traditional Rural Biotopes and grasslands managed via Agri-environment Scheme (B, D, F). All nine different simulation settings included 100 replicate runs. Species parameterisation: BASE = the default model parameterisation (K = 250; DL = 3000 m; DP = 0.90; GR = 2.0); alternative values for mean distance of long-distance dispersal events (DL1 = 1500 m, DL2 = 5000 m), probability of short-distance events (DP1 = 0.80, DP2 = 0.95), growth rate (GR1 = 1.5, GR2 = 2.5) and carrying capacity (K1 = 200, K2 = 300).

Varying the four life-history parameters had notable impacts on the projected number of individuals, number of occupied grid cells and the maximal latitudinal range shift of *Maniola jurtina*. In the analysis where the default parameter values and the CORINE land cover data were used, the projected mean (± standard deviation) number of *Maniola jurtina* individuals was 1,913,603±30,162 individuals after 50 years. The strongest change in this baseline result occurred when changing the mean length for long-distance dispersal (Figure 4A). In contrast, the corresponding results from the simulations based on the more detailed land cover data, i.e. the AES-based managed grasslands and the National Survey grasslands, indicated highest importance for carrying capacity and growth rate (Figure 4B). However, the most striking result was the notable difference in the projected number of individuals in the simulations based on the two land cover data sources: in the simulations with the default demographic parameters 1,913,603±30,162 (CORINE) vs. 144,264±17,156 (AES-National Survey) individuals.

The impact of varying the four life-history traits on the projected total number of occupied 200×200 m grid cells was qualitatively similar in CORINE-based vs. AES-National Survey data based simulations. Here, the largest life-history trait based impact was related to alternative carrying capacities (Figure 4C and 4D), and the quantitative difference in the number of occupied cells between simulations based on the two land cover data sources was clear, reflecting the conspicuous difference in amount of suitable habitat between the two landscape maps.

**Figure 5:**
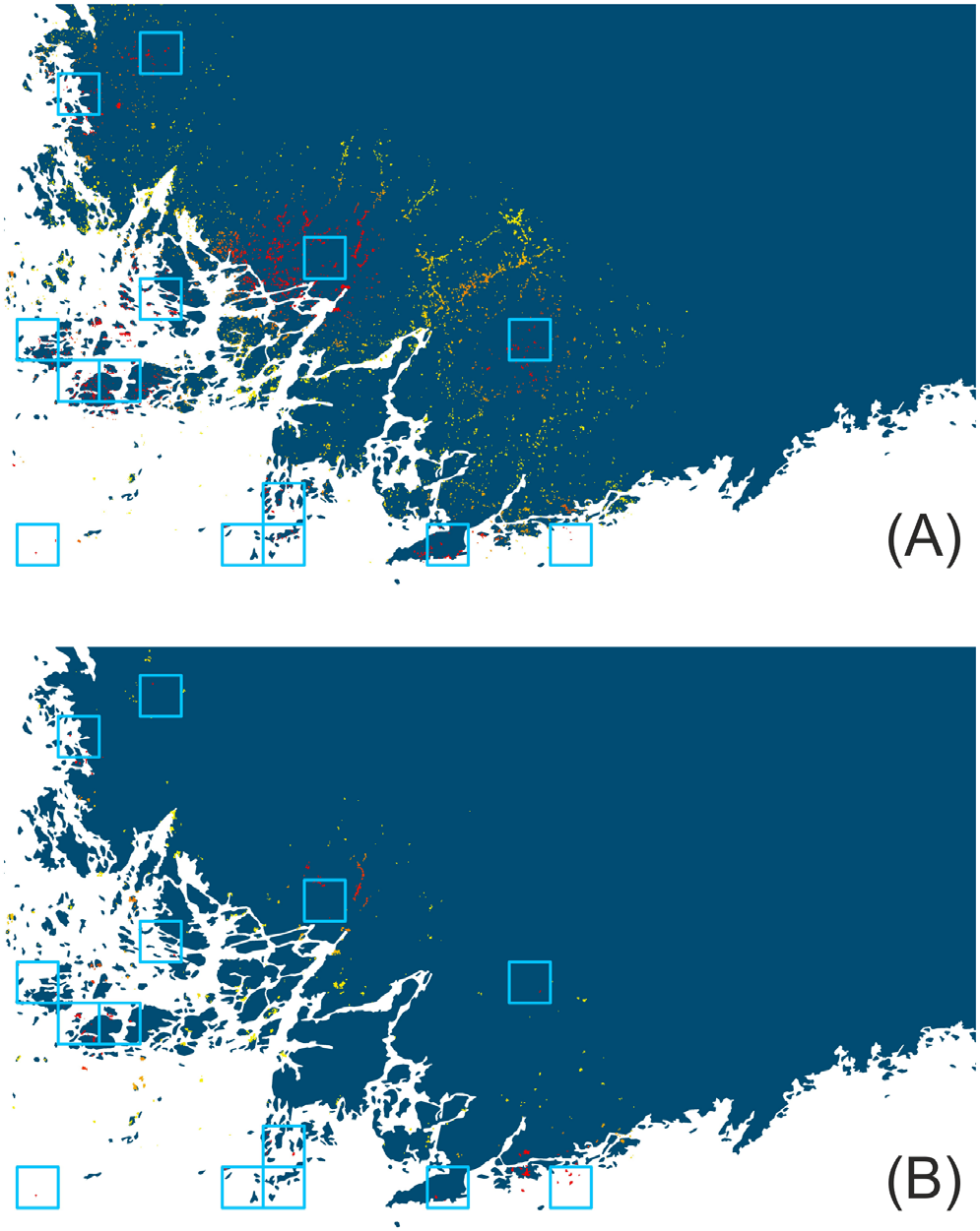
Example output maps for the simulated range expansion of *Maniola jurtina* in SW Finland. The maps for range expansion were produced by RangeShifter. Probability of a 200×200 m grid cell to be occupied after a 50-year simulation run is depicted with a colour ramp from red (high) to orange (intermediate) and yellow (low), with areas in dark blue having a probability of zero. Probability of a cell having a population was assessed based on 100 replicate simulations. Light blue squares indicate 10×10 km grid cells where the simulations were seeded. Simulations were done using default values for species traits and (A) CORINE data and (B) AES – National Survey data.

The mean (± s.d.) projected latitudinal range shift was 12.7±7.2 km in the simulations based on the CORINE data, and 3.1±4.1 km in the National Survey – AES data based simulations, respectively (Figure 4E and 4F). The largest range shifts were observed for the higher carrying capacity, but also increasing the growth rate and the probability and mean distance of long-distance dispersal events caused an increase in projected latitudinal range shifts. In very few cases, the maximal range shifts obtained exceeded 50 km in the CORINE data based results. Figure 5 shows the spatial differences in the projected occupancy probability of the 200×200 m cells in the SW coastal area of Finland. In this comparison there are clear spatial differences between the model outputs from CORINE data vs. National Survey – AES data, whereas the corresponding differences stemming from varying the four species traits were more subtle (results not shown).

### Issoria lathonia – the grassland generalist

**Figure 6:**
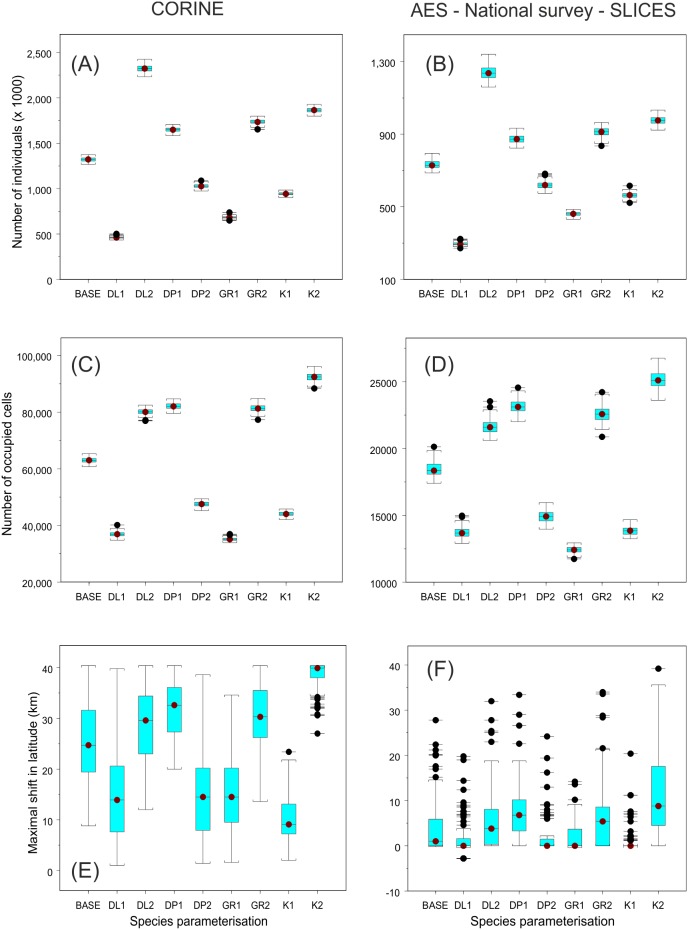
The projected total abundance and range dynamic statistics for *Issoria lathonia.* The projected total number of *Issoria lathonia* individuals (A–B), number of 200×200 m cells occupied (C–D) and maximal range shift of the butterfly (E–F) at the end of a 50 year dynamic simulation period. Simulations were conducted using either summed cover of CORINE classes ‘Pastures’ and ‘Natural grassland’, ‘Land principally occupied by agriculture, with significant areas of natural vegetation’, ‘Abandoned arable land’ and field margins (A, C, E) vs. open grasslands included in the National Survey, SLICES database and those managed via AES (B, D, F). For species parameterisation see Figure 3.

The corresponding simulations for *Issoria lathonia* showed qualitatively similar patterns (Figure 6). There was a substantial quantitative difference between the results obtained for the different landscape datasets. The projected number of individuals and occupied cells were clearly higher in the CORINE results (mean ± s.d.): 1,144,264±23,470 vs. 733,457±24,782 individuals, and 62,988±993 vs. 18,444±574 occupied 200×200 m cells, in CORINE data vs. AES – National Survey – SLICES data based results, respectively (Figure 6, simulations with default values).

**Figure 7:**
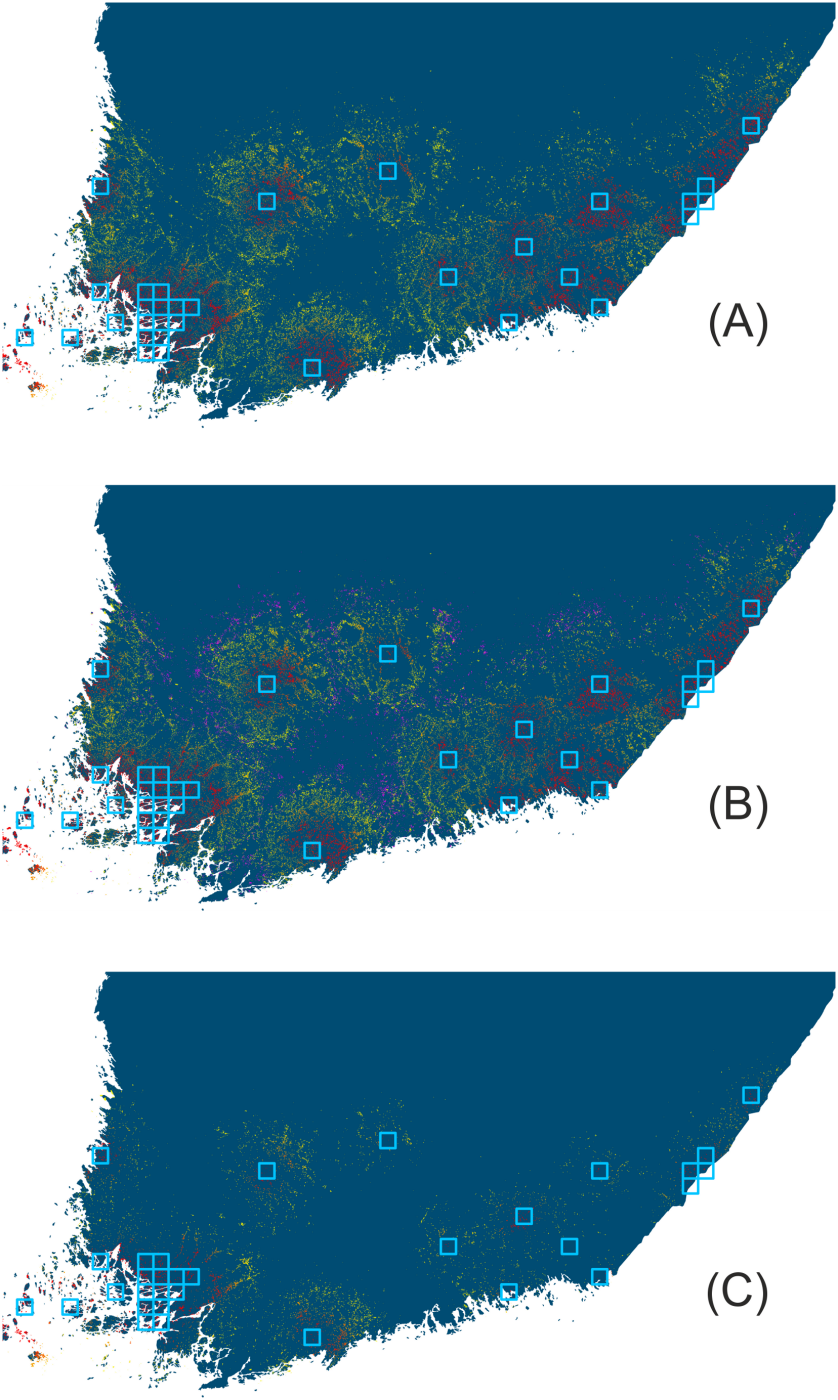
Example output for the simulated range expansion of *Issoria lathonia* in S Finland. Probability of a 200×200 m grid cell to be occupied after a 50-year simulation run is depicted with a colour ramp from red (high) to orange (intermediate) and yellow (low), with areas in dark blue having a probability of zero. Simulations were done using (A) CORINE data and default values for species traits, (B) CORINE data and increased carrying capacity value, and (C) AES – National Survey – SLICES data and default values for species traits. In (B), areas in pink indicate 200×200 m cells projected to have a population only when the higher carrying capacity is assumed.

The differences between the projected maximal range shift between the two land cover data types were also prominent (Figure 6E and 6F). In the CORINE data based results, mean range shifts were often projected to be larger than 20 km, whereas in the AES – National Survey – SLICES data based results only a limited number of individual runs exceeded a shift of 20 km. In both land cover data sets, higher carrying capacity returned the largest projected shifts. These differences are visible in the maps of species’ occupancy probabilities in the simulations based on CORINE data and default trait parameters vs. increased carrying capacity (Figure 7A and B), particularly as the extended expansion of range margin in certain regions. However, overall the spatial differences are more notable when probability of occupancy values from simulations based on CORINE data are compared with those based on AES – National Survey – SLICES data (Figure 7A and C).

### Main traits affecting range expansion

To illuminate which of the four studied life-history traits had the strongest impact of the three measures of the species’ range and population dynamics we summarized the most influential traits for the study species in Table 1. This summary shows that altering the carrying capacity typically has the strongest impact, especially on mean maximal range shifting, while the second most important driver is long-distance dispersal ability.

**Table 1:** Summary of the varied life-history traits causing largest change in the three measured species’ range expansion measures, i.e. the projected number of butterfly individuals, number of occupied 200×200 m grid cells and mean maximal latitudinal range shift, shown for the two species and two land cover types separately.

	*Maniola jurtina*		*Issoria lathonia*	
Number of individuals(x 1000)	CORINE data	AES – NationalSurvey data	CORINE	AES – National Survey –SLICES data
Lowest	DL1 (1,495±22)	K1 (67±13)	DL1 (461.7±12)	DL1 (296±9.8)
Highest	DL2 (2,350±44)	K2 (222±31)	DL2 (2,324. ±39)	DL2 (1240±39)
**Number of occupied 200-m grid cells**				
Lowest	K1 (10,283±143)	K1 (416±58)	GR1 (35,132±591)	GR1 (12426±243)
Highest	K2 (16,673±360)	K2 (1,242±146)	K2 (92,356±1,573)	K2 (25124±658)
**Maximal latitudinal range shift**				
Lowest	K1 (6.3±2.2)	K1 (1.0±1.5)	K1 (10.0±4.3)	DL1 (1.6±4.1)
Highest	K2 (24.1±9.8)	K2 (5.9±7.6)	K2 (38.4±3.0)	K2 (12.0±10.9)

The mean value (+/− standard deviation) from the 100 replicate runs is given for each measure in parenthesis. Species parameterisation abbreviations: *Maniola jurtina* and *Issoria lathonia*: DL1 = 1500 m, DL2 = 5000 m (alternative values for mean distance of long-distance dispersal events); K1 = 200, K2 = 300 (alternative values for carrying capacity); *Issoria lathonia*, GR1 = 1.5 (lower alternative value for population growth rate).

## Discussion

### Land cover data and species habitat specificity

We have shown that the selection of the land cover data upon which dynamic models are built may have a major effect on the projections of species’ range expansion. These findings are important because systematic land cover data from detailed field surveys rarely exist for larger regions. CORINE is one of the few systematically constructed land cover databases covering continent-wide areas and it is commonly used in species distribution modelling [51] [51], [52], [69]. However, its usefulness with respect to modelling species with strict habitat requirements is insufficiently known [20]. Several niche modelling studies have shown that the projections of species distributions may be substantially affected by the selection of environmental variables, including land cover variables [70], [71]. The results from our study illustrate the fact that using insufficient quality landcover data can introduce substantial bias also into the results of dynamic modelling exercises projecting species responses to climate change.

The differences in the projected species population abundance and range dynamics gained using the AES – National Survey data based simulations compared to those based on CORINE data emerge from certain important sources. The quality of the grassland sites in the National Survey of Rural Biotopes and those managed for biodiversity via AES specific contracts very likely varies less than the CORINE data from the perspective of grassland specialist butterflies which often require managed unimproved grasslands [72]–[74]. However, spatial cover of more thorough field investigations (including National Survey of Valuable Traditional Rural Biotopes) is often constrained by the limited resources, which may result in the underestimation of habitat availability in insufficiently surveyed areas. This is likely to be one of the reasons behind the very substantial differences in estimated habitat availability in the SW archipelago. In addition, here uptake of AES contracts may also be lower than in the mainland areas. In contrast, the spatial cover of CORINE data is better, but it may be more variable and overestimate the habitat availability, especially for grassland specialists. This is because CORINE data are based on various sources such as other existing land cover databases and satellite imagery.

The recorded occurrences of *Maniola jurtina* in SE Finland are a point of specific interest. Namely, there are some isolated 10×10 km grid cells with records of the species in 2001–2011 that are situated far from the potential source populations (Figure 1). To reach these cells from the earlier known sites would require dispersal over several tens of kilometres within ten years, a situation hardly possible only via the network of AES – National Survey grasslands, as shown by our simulation results. Three factors may play a role here. First, the map of known records for *Maniola jurtina* is inevitably an underestimation of the true distribution because of the spatial variation in survey effort [see 75,76]. Second, it is possible that the grassland network in SE Finland is in many areas insufficient to maintain longer-term populations of grassland specialists and thus regional butterfly populations may be dependent on the constant arrival of immigrants from Russia, where higher quality grasslands are more common, possibly representing a large-scale source-sink system between Russia and Eastern Finland [73]. Thirdly, it is possible that the habitat fidelity of *Maniola jurtina* is in a changing stage. Thus, we may be dealing with changing habitat specificity of a species at its northern range boundary where climate has recently become more favourable. Due to this, the butterfly might now utilise a wider range of grassland habitats than earlier [41].

### Impact of uncertainty in life-history traits relative to uncertainty in land cover

As the climate impacts research community increasingly uses dynamic models to project species’ future distributions, it is crucial that we begin to gain some insight into the relative importance of different forms of input uncertainty to the uncertainties associated with the outputs from these models. We ran our dynamic model using an illustrative set of values for key life history parameters that were chosen to represent the bounds of uncertainty for those parameters. This allows us to compare how influential uncertainty is in each of those life history parameters as well as comparing how important uncertainty around life history generally is relative to that due to choice of landscape database.

Unsurprisingly, altering the four focal life-history traits had an impact on the projected number of individuals and occupied grid cells of the two study species. Earlier studies have indicated that assuming a higher dispersal ability will allow a faster range expansion and a more successful tracking of changing climate [13], [25] and similar results are obtained in our study with more rapid range expansions when the probability of the long-distance dispersal jumps is increased. However, our results are not fully straightforward and the impact of increasing long distance dispersal depends on the landscape context. With *Maniola jurtina*, increasing the mean magnitude of long-distance dispersal events had the strongest impact on projected number of individuals within the CORINE pastures network. Otherwise, the strength of this effect was much reduced. With *Issoria lathonia*, increasing the mean length of long-distance movements typically had a more prominent role than increasing the proportion of long distance dispersal and showed consistent effects on both landscape maps. Increasing the probability of long-distance dispersal events had mainly an intermediate impact on the projected population estimates. This effect was larger than that of the length of long-distance movements for the species’ maximal range shifts, being most evident in the results for *Issoria lathonia* and CORINE grassland data.

As expected [77], we found population growth rate impacted the projected population dynamics during range expansion. Theoretical models [5], [40] have also highlighted the importance of rapid growth rate for species population persistence under a changing climate. Further, in one study very relevant in the context of our work, Willis et al. [16] showed that the projected rate of expansion of the *Pararge aegeria* butterfly in the UK was especially sensitive to altering population dynamics; a 25% increase in productivity resulted in a 56% increase in range expansion.

Interestingly, we found very substantial sensitivity to uncertainty in carrying capacity showed for both *Maniola jurtina* and *Issoria lathonia*. Classical theory on range expansion has typically stressed the important joint roles of population growth rate and dispersal [67], [77]–[79] and has not highlighted an important role for carrying capacity. However, South [24] showed with a spatially explicit population model that there may be complicated links between dispersal success, dispersal initiation mechanism, patch growth rate and patch carrying capacity, which all ultimately affect population persistence. Moreover, a recent theoretical study by Bocedi et al. [80] using artificial fragmented landscapes and theoretical species, demonstrated that carrying capacity can often be influential. The results we present here lend weight to the suggestion that, at least on fragmented landscapes, we need to pay greater attention to this parameter.

However, it should be noted that what our result highlight most strongly is the substantial uncertainty we find due to the choice of habitat maps relative to the uncertainties due to the demographic parameters. In dynamic modelling studies there is often some discussion of potential uncertainties related to estimation of demographic parameters and especially dispersal, but much less attention is given to uncertainty in the spatial representation of suitable habitat. Our results demonstrate very clearly that uncertainty due to habitat mapping can be at least as great as that due to the demographic parameters. This reinforces the results of a study by Willis et al. [16] that demonstrated that the projected rate of spread on *Pararge aegeria* butterfly was more sensitive to altering habitat availability than variation in demographic factors and seed locations in the simulations. A further study focussing on population viability rather than range expansion [12] has specifically focused the importance of the uncertainties in developing habitat maps for a species, such as potential errors in satellite imagery and georeferencing. They used a spatially explicit model based on two contrasting habitat maps created from remote imagery for a forest dwelling bird species, a ‘generous’ and ‘strict’ habitat map. The selection between the two habitat maps caused differences in total population size three times more important than other factors, such as dispersal model type, maximal dispersal distance and bird clutch size. Our study bridges these previous two studies by exploring the extent of uncertainty that arises in projections of range expansion due to the choice of dataset for constructing a habitat map. Interestingly, our results also highlight that the extent of uncertainty due to choice of dataset can be very different between species; we found a much greater effect for *Maniola jurtina* than for *Issoria lathonia.*

### Implications for conservation planning

Our modelling results have clear importance for conservation planning because conservation biologists and managers are currently seeking robust tools to project the changes in species’ distributions in response to changing climate. Dynamic range expansion models are considered one promising approach to develop improved assessments on the impacts of global changes, potentially providing a sounder basis to allocate the scarce conservation resources than the widely applied bioclimatic envelope models [11]. However, the end-users of dynamic models need to be aware of the limitations in the modelling approaches available. Indeed, our results suggest that dynamic modelling approaches should also be used with caution when applied to real-life nature conservation questions. This is because dynamic range expansion and population models have sources of uncertainty of their own and failing to acknowledge this may invoke a false sense of confidence [12], [17], [18].

Such uncertainties are centred around three main issues: (1) the scarcity of accurate species and habitat data over larger areas [15], (2) uncertainties in data for species life-history traits critical for dispersal and population dynamics [11], [22], and (3) difficulties in determining direct and robust links between the species’ habitat requirements and the land cover data available [12]. Under these circumstances, running sensitivity analysis for dynamic species population models before they are used in different applied conservation and management planning questions is essential [18]. The results of this study demonstrate that the sensitivity analysis can indeed provide important insights for the sensitivity of dynamic models to altering species parameters and habitat requirements.

Developing improved conservation planning tools for grassland species is important, because different types of grassland habitats from unimproved semi-natural grasslands to non-cultivated elements such as larger field margins and wooded pastures have faced a drastic decline during the last century [30], [34]. This has resulted in a major loss of landscape heterogeneity and habitats favoured by many butterfly species. Our simulation results suggest that the possibilities of both grassland habitat specialist and generalist butterfly species to adapt to projected climate change may be limited. Only in our most generous simulation setting, *Issoria lathonia* in network of CORINE grasslands and abandoned cultivated land and field margins, the mean maximal range shifts were projected to exceed 20 or 30 km kilometres within 50 years. These forecasts are well in line with the recently observed range shifts of grassland butterflies in Finland. Interestingly, they fall much below the observed fastest range shifts in butterflies, generally encountered in forest edge generalist species with high dispersal ability [28].

Moreover, many grassland butterfly species depend on the occurrence of semi-natural grasslands managed by mowing or grazing [73], [74], or other similar higher-quality grasslands connected with traditional agricultural practices [81]. Our simulation results for *Maniola jurtina* suggest that the ecological sufficiency of the grasslands included in the National Survey of Rural Biotopes and those managed by specific AES is poor for a strict grassland specialist species depending on high-quality sites. In particular, the likelihood that the grassland specialist species respond to climate change by dispersing into new suitable areas in southern Finland seems low, as the projected maximal range shifts for *Maniola jurtina* were modest at best.

In conclusion, the adaptation and persistence possibilities of grassland specialist species under environmental changes in our study area appear to be very limited; thus, major changes are required to improve the critical situation of these habitats and their species. The two complementary main lines of required future action are: (1) increasing of the area of grasslands managed for biodiversity to mitigate long-term habitat loss impacts [82] and support local population persistence [83], and (2) improving their connectivity to support grassland species range shifting across the landscape [5].

## Supporting Information

Figure S1 **Shape of emigration probability curve used in the simulations.** The calculation of the emigration probability curve is based on the density-dependent emigration assumption with maximum dispersal probability D_0_ = 0.4, slope α = 5.0 and inflection point β = 1.0.
(TIF)

Figure S2 **The cover of suitable grassland habitat for** ***Issoria lathonia*** **, a grassland generalist butterfly, in two exemplary 10×10 km grid cells based on two different sources of spatial grassland data.** A and C: summed cover of all kinds of open grassland included in the National Survey, AES and the SLICES database in each of the 200×200 m cells; B and D: summed cover of CORINE classes ‘Pastures’, ‘Natural grassland’, ‘Land principally occupied by agriculture, with significant areas of natural vegetation’, and ‘Abandoned arable land’, together with the cover of field margins, in each of the 200×200 m cells.
(TIF)

Text S1 **Supporting information for the two model species’ parameterisation for the RangeShifter dynamic range expansion simulations.** More in-depth description of the process how the dispersal and population biological parameters for the two butterfly species, *Maniola jurtina* and *Issoria lathonia*, required to perform the range expansion simulations with RangeShifter algorithm were done based on the following sources: an extensive literature search, the data extracted from long-term butterfly monitoring surveys carried out in Finland such as the Finnish Butterfly Monitoring Scheme, published data on population biological parameters from studies on ecologically similar species, and empirical data -based expert assessments on the general variation of demographic parameters among grassland butterfly species.
(DOCX)
