# Supplementary figures and images for: Impacts of Land Cover Data Selection and Trait Parameterisation on Dynamic Modelling of Species’ Range Expansion
Source: PLoS One. 2014 Sep 29;9(9):e108436. doi: 10.1371/journal.pone.0108436 (PMC4180940; doi:10.1371/journal.pone.0108436)

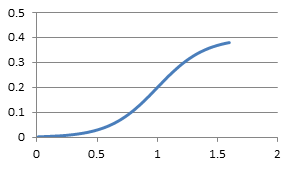

Supplement: Figure S1 — Shape of emigration probability curve used in the simulations. The calculation of the emigration probability curve is based on the density-dependent emigration assumption with maximum dispersal probability D0 = 0.4, slope α = 5.0 and inflection point β = 1.0. (TIF) [file pone.0108436.s001.tif]

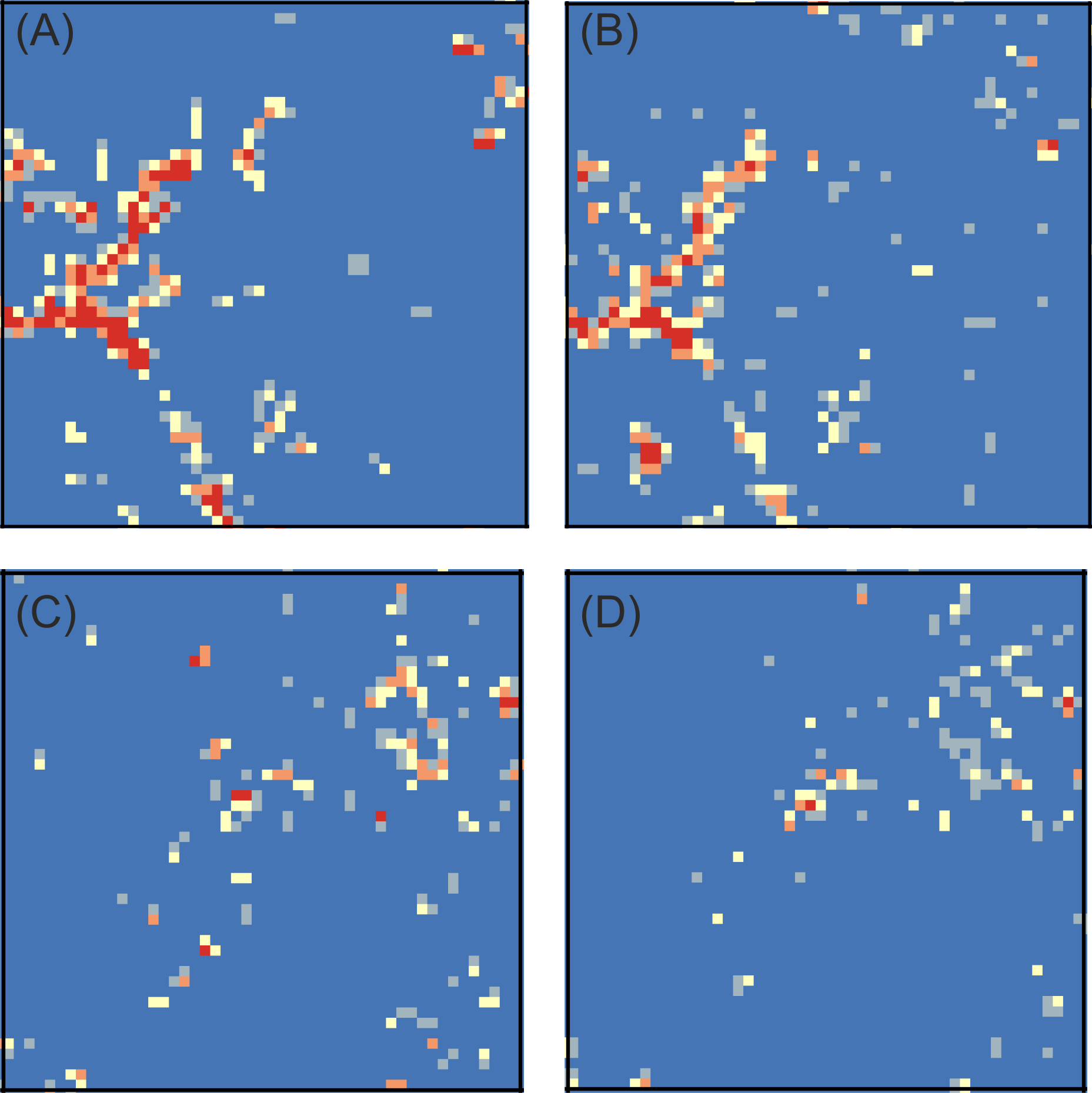

Supplement: Figure S2 — The cover of suitable grassland habitat for Issoria lathonia , a grassland generalist butterfly, in two exemplary 10×10 km grid cells based on two different sources of spatial grassland data. A and C: summed cover of all kinds of open grassland included in the National Survey, AES and the SLICES database in each of the 200×200 m cells; B and D: summed cover of CORINE classes ‘Pastures’, ‘Natural grassland’, ‘Land principally occupied by agriculture, with significant areas of natural vegetation’, and ‘Abandoned arable land’, together with the cover of field margins, in each of the 200×200 m cells. (TIF) [file pone.0108436.s002.tif]
