# Supplementary material for: Impacts of Land Cover Data Selection and Trait Parameterisation on Dynamic Modelling of Species’ Range Expansion
Source: PLoS One. 2014 Sep 29;9(9):e108436. doi: 10.1371/journal.pone.0108436 (PMC4180940; doi:10.1371/journal.pone.0108436)
Supplement: Text S1 — Supporting information for the two model species’ parameterisation for the RangeShifter dynamic range expansion simulations. More in-depth description of the process how the dispersal and population biological parameters for the two butterfly species, Maniola jurtina and Issoria lathonia, required to perform the range expansion simulations with RangeShifter algorithm were done based on the following sources: an extensive literature search, the data extracted from long-term butterfly monitoring surveys carried out in Finland such as the Finnish Butterfly Monitoring Scheme, published data on population biological parameters from studies on ecologically similar species, and empirical data -based expert assessments on the general variation of demographic parameters among grassland butterfly species. (DOCX) [file pone.0108436.s003.docx]

**Text S1. Supporting information for the two model species’ parameterisation for the RangeShifter dynamic range expansion simulations.** More in-depth description of the process how the dispersal and population biological parameters for the two butterfly species, *Maniola jurtina* and *Issoria lathonia*, required to perform the range expansion simulations with RangeShifter algorithm were done based on the following sources: an extensive literature search, the data extracted from long-term butterfly monitoring surveys carried out in Finland such as the Finnish Butterfly Monitoring Scheme, published data on population biological parameters from studies on ecologically similar species, and empirical data -based expert assessments on the general variation of demographic parameters among grassland butterfly species.

Several empirical studies on *Maniola jurtina* [e.g. 1,2,3] were found but corresponding information was largely lacking for *Issoria lathonia*. Thus for *Issoria*, following earlier studies [4,5], parameter values were partly determined based on studies on ecologically similar species and how the demographic parameters have been observed or estimated to vary among grassland butterfly species more generally. For both species, expert-knowledge-based assessments on the study species’ biology were used to adjust the life-history parameters. For *Issoria lathonia*, carrying capacity was estimated using transect count data from the Finnish Butterfly Monitoring Scheme [6], and for *Maniola jurtina* using additional data from the Åland Islands in south-western Finland [7]. Only observations from butterfly transects placed in grassland habitats were used. The sampling effort was standardized by using an equal-sized sample of seven counts for each transect and year. Carrying capacity for both *Maniola jurtina* and *Issoria lathonia* was calculated as an average density for the highest 10% of observed butterfly densities (individuals per 1 km of transect 5 m in width).

For the four focal parameters, i.e. carrying capacity, maximum potential population growth rate, mean dispersal distance and probability of long-distance dispersal events, an intermediate ‘default’ value and lower and higher alternative values were determined, providing a sensitivity analysis for these parameters. The data from the Finnish Butterfly Monitoring Scheme indicated that carrying capacity for *Maniola jurtina* was 232 individuals per 1 km of transect, and for *Issoria lathonia* 29 individuals, respectively. This difference in maximum observed densities between the two species is apparent also in butterfly monitoring data from other countries, e.g. the Netherlands [8]. However, for *Issoria lathonia*, densities twice as high as those recorded in Finland have been recorded in the Netherlands [8]. Thus, we selected carrying capacity K = 250 individuals/ha as the default value and K = 200 and K = 300 as alternatives for *Maniola jurtina*, and K = 60 individuals/ha as the default value and K = 30 and K = 90 as alternatives for *Issoria lathonia*, respectively.

There were no estimates on growth rate available for our study species. However, studies on other butterfly species have suggested that growth rates can vary considerably between different species and localities. Baguette and Schtickzelle [9] showed that R_max_ may range from values less than 1.3 in slowly growing populations (*Boloria aquilonaris*) to over 3.1 in species with a very rapid population growth capacity (*Euphydryas aurinia*). Expert judgements based on field observations suggest that both *Maniola jurtina* and *Issoria lathonia* are likely to show intermediate population growth rates. Thus, we opted for using R_max_ = 2.0 as the default value and R_max_ = 1.5 and R_max_ = 2.5 as the two alternatives for both species [cf. 10].

For the dispersal kernels, we used double negative exponential [11,12] which allows setting the parameters separately for the more common short-distance dispersal events (‘dispersal I’) and the rarer long-distance dispersal events (‘dispersal II’). For both species, only one value was used for the mean short-distance dispersal whereas values for the latter parameter were varied. Reviewed literature as well as expert-based judgements indicated that *Issoria lathonia* is more mobile than *Maniola jurtina* [13-16]. For *Maniola jurtina*, results from mark-recapture studies [1,3,15,17,18] suggest that the overall mean dispersal distances vary between 100 and 300 meters. Consequently, we used 150 m as the selected value for the mean short-distance dispersal for *Maniola jurtina*, and 300 m for the more mobile *Issoria lathonia*. Öckinger and Smith [15] reported that a number of marked *Maniola* individuals dispersed more than 1 km, with few individuals moving almost 3 km. However, the National Butterfly Recording Scheme in Finland –database includes records of *Maniola jurtina* from one isolated island (Utö) in the archipelago of SW Finland. The distance from Utö to the nearest larger island with suitable habitat is ca. 10 km, suggesting that the species may occasionally migrate over considerable distances [cf. 19]. Given these background data, we used 3 km as the intermediate default value for the mean long-distance dispersal (dispersal II), and 1.5 km and 5 km as alternatives for *Maniola jurtina*. To acknowledge the higher mobility of *Issoria lathonia*, we used values 3 km, 5 km and 10 km for it, respectively.

For assessing the probability of long-distance dispersal events we used the observation of Öckinger and Smith [15] on the 11.7 % of the marked *Maniola* individuals dispersing more than 1 km as a template. For both of our study species we set the probability of dispersal kernel I in the RangeShifter as either 0.80, 0.90 (default) or 0.95 (i.e. 90 % of the individuals moving following short-distance dispersal kernel, 10 % long-distance dispersal kernel as default, and so forth).

The remaining model parameters required to run the simulations with RangeShifter were determined as follows: we used (i) asexual / only female model, as in butterflies it is enough to establish a new population when a mated female disperses to a new site, (ii) one reproductive season per year [cf. 20], (iii) competition coefficient (b) as 1.0 providing for compensatory density dependence, (iv) assumption of ‘gently S-shaped’ (slowly rising and then levelling off) density-dependent emigration (see Figure S2) where the three parameters affecting the shape of emigration probability curve were set as maximum dispersal probability D_0_ = 0.4, slope α = 5.0 and inflection point β = 1.0 [cf. 21,22], (v) dispersal mortality = 0.0, and (vi) no environmental stochasticity was assumed to take place. Finally, as the settlement rule we let the individuals landing at an unsuitable 200-m grid cell to randomly choose one of the suitable 8 neighbouring cells, or die if none of them were suitable. Note that the dispersal mortality parameter controls the likelihood that an emigrating individual dies in transit. Even when this parameter is set to zero, some (potentially many) emigrants suffer mortality due to failing to successfully arrive at suitable habitat.

We opted for using the assumption of no environmental stochasticity because our focus was in examining the potential impacts of the four key species life-history traits on simulation results and to assess the role that demography and representation of the landscape play in determining spread rates. However, it should be noted that RangeShifter inherently incorporates two key sources of stochasticity. The first is classic demographic stochasticity that is implemented by each individual producing a number of offspring that is drawn at random from a Poisson distribution. The second source of stochasticity is in the dispersal phase; the direction and distance that each emigrant travels are both determined at random. These sources of stochasticity can both be influential in determining range dynamics especially at expanding margins with low population density [23,24].

**References**

1. Dover JW, Clarke SA, Rew L (1992) Habitats and movement patterns of satyrid butterflies (Lepidoptera: Satyridae) on arable farmland. Entomol Gazette 43: 29-43.

2. Grill A, Schtickzelle N, Cleary DFR, Nève G, Menken SBJ (2006) Ecological differentiation between the Sardinian endemic Maniola nurag and the pan-European Maniola jurtina. Biological Journal of the Linnaean Society 89: 561-574.

3. Ouin A, Martin M, Burel F (2008) Agricultural landscape connectivity for the meadow brown butterfly (Maniola jurtina). Agriculture, Ecosystems & Environment 124: 193–199.

4. Hanski I (1994) A practical model of metapopulation dynamics. Journal of Animal Ecology 63: 151-162.

5. Hill JK, Collingham YC, Thomas CD, Blakeley DS, Fox R, et al. (2001) Impacts of landscape structure on butterfly range expansion. Ecology Letters 4: 313-321.

6. Heliölä J, Kuussaari M, Niininen I (2010) Maatalousympäristön päiväperhosseuranta 1999–2008. (Butterfly monitoring in Finnish agricultural landscapes 1999–2008). The Finnish Environment 2/2010: 1-65.

7. Schulman A, Heliölä J, Kuussaari M (2005) Farmland biodiversity on the Åland islands and assessment of the effects of the agri-environmental support scheme. The Finnish Environment 734: 1-210.

8. van Swaay CAM (2003) Butterfly densities on line transects in The Netherlands from 1990-2001. Entomologische Berichten 63: 82-87.

9. Baguette M, Schtickzelle N (2006) Negative relationship between dispersal distance and demography in butterfly metapopulations. Ecology 87: 648-654.

10. del Barrio G, Harrison PA, Berry PM, Butt N, Sanjuan ME, et al. (2006) Integrating multiple modelling approaches to predict the potential impacts of climate change on species' distributions in contrasting regions: comparison and implications for policy. Environmental Science & Policy 9: 129-147.

11. Hovestadt T, Binzenhofer B, Nowicki P, Settele J (2011) Do all inter-patch movements represent dispersal? A mixed kernel study of butterfly mobility in fragmented landscapes. Journal of Animal Ecology 80: 1070-1077.

12. Nathan R, Klein E, Robledo-Arnuncio JJ, Revilla E (2012) Dispersal kernels: review. In: Clobert J, Baguette M, Benton TG, Bullock J, editors. Dispersal Ecology and Evolution: Oxford University Press. pp. 187–210.

13. Komonen A, Grapputo A, Kaitala V, Kotiaho J, Päivinen J (2004) The role of niche breadth, resource availability and range position on the life history of butterflies. Oikos 105: 41-54.

14. Maes D, Bonte D (2006) Using distribution patterns of five threatened invertebrates in a highly fragmented dune landscape to develop a multispecies conservation approach. Biological Conservation 133: 490-499.

15. Öckinger E, Smith HG (2007) Asymmetric dispersal and survival indicate population sources for grassland butterflies in agricultural landscapes. Ecography 30: 288-298.

16. Öckinger E, Smith HG (2008) Do corridors promote dispersal in grassland butterflies and other insects? Landscape Ecology 23: 27-40.

17. Grill A, Cleary DFR, Stettmer C, Brau M, Settele J (2008) A mowing experiment to evaluate the influence of management on the activity of host ants of Maculinea butterflies. Journal of Insect Conservation 12: 617-627.

18. Schneider C (2003) The influence of spatial scale on quantifying insect dispersal: an analysis of butterfly data. Ecological Entomology 28: 252–256.

19. Mitikka V, Heikkinen RK, Luoto M, Araujo MB, Saarinen K, et al. (2008) Predicting range expansion of the map butterfly in Northern Europe using bioclimatic models. Biodiversity and Conservation 17: 623-641.

20. Pöyry J, Leinonen R, Soderman G, Nieminen M, Heikkinen RK, et al. (2011) Climate-induced increase of moth multivoltinism in boreal regions. Global Ecology and Biogeography 20: 289-298.

21. Nowicki P, Vrabec V (2011) Evidence for positive density-dependent emigration in butterfly metapopulations. Oecologia 167: 657-665.

22. Bocedi G, Palmer SCF, Pe’er G, Heikkinen RK, Matsinos Y, et al. (2014) RangeShifter: a platform for modeling spatial eco-evolutionary dynamics and species’ responses to environmental changes. Methods in Ecology and Evolution, Accepted article. doi: 10.1111/2041-210X.12162.

23. Clark JS, Lewis M, Horvath L (2001) Invasion by extremes: population spread with variation in dispersal and reproduction. The American Naturalist 157: 537-554.

24. Travis JMJ, Harris CM, Park KJ, Bullock JM (2011) Improving prediction and management of range expansions by combining analytical and individual-based modeling approaches. Methods in Ecology and Evolution 2: 477-488.
